# EHMTI-0228. A clinical interview versus prospective headache diaries in the diagnosis of menstrual migraine without aura

**DOI:** 10.1186/1129-2377-15-S1-D68

**Published:** 2014-09-18

**Authors:** KG Vetvik, EA MacGregor, AC Lundqvist, MB Russell

**Affiliations:** 1 Institute of Clinical Medicine and Head and Neck Research group Research Centre, University of Oslo and Akershus University Hospital, Lorenskog, Norway; 2 Barts and the London School of Medicine and Dentistry, Centre for Neuroscience and Trauma Blizard Institute, London, UK; 3 Head and Neck Research Group and HØKH Research Centre and Institute of Clinical Medicine and Department of Neurology, Akershus University Hospital and University of Oslo, Lørenskog, Norway; 4 Head and Neck Research Group Research Centre and Institute of Clinical Medicine, Akershus University Hospital and University of Oslo, Lørenskog, Norway

## Introduction

The International Classification of Headache Disorders (ICHD) II and III beta defines menstrual migraine as attacks of migraine without aura occurring on day 1±2 of the menstrual cycle in ≥2/3 menstruations. According to the ICHD III beta, a three month prospective headache diary is required in order to establish the diagnosis of MM.

## Aims

To compare the diagnosis of MM from a clinical interview based on the ICHD II-criteria for MM, with prospective headache diaries in a population-based study.

## Methods

237 women with self-reported migraine in at least half of menstruations were interviewed by a neurologist about headache and diagnosed according to ICHD II. Additionally, the MM-criteria were expanded to include other types of migraine related to menstruation. Subsequently, all women were asked to complete three month prospective headache diaries.

## Results

A total of 123 (52%) women completed both clinical interview and diaries. Thirty-eight women were excluded from the analyses; two had incomplete diaries and 36 women recorded £1 menstruation, leaving 85 diaries eligible for analysis. Sensitivity, specificity, positive and negative predictive value and Kappa for the diagnosis of MM in clinical interview vs. headache diary were 82%, 83%, 90%, 71% and 0.62 (95% CI 0.46-0.80). Using a broader definition of MM, Kappa was 0.64 (95% CI 0.47-0.83).

## Conclusion

A thorough clinical interview is valid for the diagnosis of MM. When this is undertaken, prospective headache diaries should not be mandatory to diagnose MM but may be necessary to exclude a chance association.

No conflict of interest.

